# Chronic TBPH Exposure Drives the Transition from Steatosis to Hepatic Fibrosis via Lipid Droplet Dysregulation in Zebrafish

**DOI:** 10.3390/biology15060463

**Published:** 2026-03-13

**Authors:** Yiming Liu, Dingxi Pan, Mingying Li, Wei Guo

**Affiliations:** 1 State Key Laboratory of Conservation and Utilization of Bio-Resources, School of Life Sciences, Center for Life Sciences, Yunnan University, Kunming 650500, China; 2 School of Ecology and Environmental Science, Yunnan University, Kunming 650500, China

**Keywords:** TBPH, MASH, hepatic fibrosis, lipid droplets, zebrafish

## Abstract

Bis(2-ethylhexyl)-2,3,4,5-tetrabromophthalate (TBPH) is a widely used novel flame retardant and an emerging ubiquitous environmental contaminant. While acute exposure disrupts lipid signaling, the long-term consequences of TBPH exposure on the progression of metabolic dysfunction-associated steatotic liver disease (MASLD) remain poorly understood. This study aimed to elucidate the chronic hepatotoxic effects of TBPH and the underlying molecular mechanisms. Adult zebrafish were exposed to environmentally relevant concentrations of TBPH for 6 weeks. Hepatic damage was assessed using histological examination, biochemical assays, and integrated proteomic and transcriptomic profiling. In vitro assays using HepG2 cells were conducted to validate cellular mechanisms of lipid droplet (LD) dynamics. Chronic TBPH exposure induced severe macrovesicular steatosis and significant hepatic fibrosis in zebrafish. Transcriptional analysis revealed that TBPH activated both lipid synthesis and fatty acid oxidation. In vitro results confirmed that TBPH stimulated DGAT2-mediated triglyceride synthesis and promoted LD expansion via ER-LD co-localization. Proteomic analysis identified a microfibril-associated protein 4 (Mfap4) associated with extracellular matrix remodeling and fibrosis. These findings demonstrate that chronic TBPH exposure acts as a potent metabolic disruptor, driving a pathological cascade from steatosis to fibrosis. This study provides a comprehensive adverse outcome pathway for TBPH-induced hepatotoxicity, highlighting its potential role in the etiology of metabolic dysfunction-associated steatohepatitis.

## Simple Summary

This study provides compelling evidence that chronic exposure to TBPH acts as a potent environmental disruptor of hepatic homeostasis, driving the progression from simple steatosis to fibrotic steatohepatitis in zebrafish. We demonstrate that TBPH disrupts the critical balance between hepatic lipid acquisition and expenditure. Mechanistically, this imbalance is mediated by dysregulated lipid droplet biogenesis leading to the formation of enlarged lipid droplets, which has been validated in vitro. This unresolved lipid burden subsequently triggers a transition toward a chronic inflammatory state and fibrosis. Moreover, we identified the significant upregulation of key fibrotic drivers in the high-concentration exposure group. Collectively, these findings elucidate a distinct mechanism where long-term TBPH exposure exacerbates liver injury beyond the capacity of metabolic adaptation.

## 1. Introduction

Bis(2-ethylhexyl)-2,3,4,5-tetrabromophthalate (TBPH) is a substitute for traditional brominated flame retardants, like penta-brominated diphenyl ethers (BDEs). Due to its extensive production and application over the past decade, TBPH has become a ubiquitous environmental contaminant, detected in the air, soil, sediments, and surface water [1,2,3], as well as in the human body [4]. While in vitro studies have suggested potential cytotoxicity [5,6], the specific metabolic consequences of TBPH exposure in vivo remain under investigation. In our recent study on zebrafish embryos, we observed that acute exposure to TBPH induced a reduction in lipid storage in larvae by promoting the demethylation of the *pparg* promoter and provoking downstream gene transcription involved in lipid metabolism [7]. These *pparg* agonist effects may contribute to the initiation of hepatic steatosis or transition from simple fatty liver to the hepatic manifestation of metabolic syndrome, such as metabolic dysfunction-associated steatotic liver disease (MASLD) [8,9].

Multiple factors, including genetic, metabolic, environmental, and gut microbial, can affect lipid and glucose metabolism, leading to hepatic fat accumulation and creating a proinflammatory environment that triggers cellular injury in the liver [10,11]. These factors collectively drive insulin resistance, oxidative stress, and lipotoxicity, creating a proinflammatory environment that promotes hepatocellular injury and fibrosis [12,13]. The global prevalence of MASLD among adults stands at around 30%. By 2019, it was estimated that there were roughly 1.66 billion prevalent cases of MASLD globally [14]. The increasing incidence rates of MASLD have emerged as a major public health issue worldwide [15,16]. Emerging evidence from murine models suggests that the dysregulation of organelle interaction, specifically the communication between the endoplasmic reticulum (ER) and mitochondria, plays a pivotal role in the transition from simple steatosis to steatohepatitis (NASH) by impairing phospholipid transfer and mitochondrial function [17,18]. Despite this, the potential for TBPH to drive these specific organelle-level dysfunctions during chronic exposure has not been explored.

Zebrafish (*Danio rerio*) are widely utilized in toxicological research due to their rapid development and high genetic conservation with humans, particularly in lipid metabolism pathways. They serve as a robust model for studying liver development and regeneration, and the etiology of steatosis [19]. Previous studies have demonstrated that environmental pollutants can induce hepatocyte vacuolation and steatosis in zebrafish through mechanisms involving oxidative stress and ER dysfunction [20,21]. To bridge the gap between acute metabolic disruption and chronic liver pathology, this study investigated the effects of chronic TBPH exposure on the adult zebrafish liver. Zebrafish were exposed to environmentally relevant concentrations of TBPH for 6 weeks. We employed a multi-dimensional approach, combining histological assessment with hepatic proteomic profiling, to elucidate the underlying mechanisms of TBPH-induced hepatotoxicity.

## 2. Materials and Methods

### 2.1. Chemicals

TBPH (CAS: 26040-51-7; >98%) was obtained from AccuStandard, Inc. (New Haven, CT, USA). DMSO (dimethyl sulfoxide; purity >99.5%) was sourced from Sigma-Aldrich (St. Louis, MO, USA). ERp57 Antibody (cat: GTX113719) was acquired from GeneTex, Inc. (Irvine, CA, USA). DAPI (cat: EN62248) was purchased from Invitrogen Corp. (Waltham, MA, USA). LipidSpot™ 488 (cat: 70065-T) was obtained from Biotium, Inc. (Fremont, CA, USA).

### 2.2. Zebrafish Maintenance and Experimental Design

Wildtype (TU strain) were obtained from the China Zebrafish Resource Center (Wuhan, China). Adult female wildtype zebrafish were maintained at 28 ± 0.5 °C and a 14 h light/10 h dark photoperiod following a previously described protocol. Zebrafish were exposed to 0 μM (Ctrl), 0.02 μM (Low), or 2 μM (High) TBPH in a 20 L tank, and the exposure water in these tanks was replaced daily. The selected exposure concentrations were established in accordance with environmentally relevant levels [22] and the experimental parameters validated in our prior studies [8,9]. Each group contained three replicates (*n* = 3). Each tank received 0.005% DMSO and the exposure solution was 1/2 replaced in two days. After 6 weeks of exposure, zebrafish were euthanized with 400 mg/L MS-222 and sacrificed for subsequent analyses. The samples were fixed in 10% neutral formaldehyde or snap-frozen in liquid nitrogen and stored at −80 °C before analysis. TBPH content in the exposure solution was measured using an Agilent 7890B/7000C GC MS/MS (Agilent Technologies, Inc., Santa Clara, CA, USA) instrument as previously described [8]. All experiments involving zebrafish were conducted in accordance with the ethical guidelines provided in the Animal Research Usage Guide by Yunnan University.

### 2.3. Histopathological Analysis

Following euthanasia, zebrafish (*n* = 9 and 3 from reach replicate tank) were randomly selected from each group for fixation in 10% neutral formaldehyde. The fixed liver tissue was dehydrated, embedded in paraffin, sectioned into 5 μm slices, and then stained with hematoxylin–eosin staining or Masson’s trichrome staining as in previous studies [23,24]. The slides were examined under an Olympus CX41 microscope (Olympus Corporation, Tokyo, Japan). The images were analyzed using ImageJ V1.8 software.

### 2.4. Cell Culture

HepG2 cells were cultured in Dulbecco’s Modified Eagle Medium (DMEM, Thermo Fisher Scientific, Waltham, MA, USA) supplemented with 10% fetal bovine serum (FBS), 1% penicillin (100 μg/mL), and streptomycin (100 μg/mL). The cells were maintained in a humidified incubator at 37 °C with 5% CO_2_. For the in vitro exposure experiments, cells were seeded into 24-well plates containing coverslips at a density of approximately 2 × 10^5^ cells/mL. Upon reaching 70% confluence, the cells were co-treated for 24 h with various concentrations of TBPH (0, 0.2, and 2 nM) (0.1% DMSO) and 200 μM oleic acid (OA; P1383, Sigma-Aldrich, St. Louis, MO, USA) conjugated to fatty acid-free bovine serum albumin (BSA; A8806, Sigma-Aldrich, St. Louis, MO, USA). Following exposure, the cells were fixed in 4% paraformaldehyde for 15 min and washed three times with PBS. Immunofluorescence staining for ERp57 was performed as previously described [25]. Lipid droplets were stained using LipidSpot™ 488 (Biotium, Inc., Fremont, CA, USA) according to the manufacturer’s instructions, and nuclei were counterstained with DAPI. Images were acquired using a ZEISS LSM 880 confocal microscope (ZEISS, Oberkochen, Germany), and subsequent analyses were performed using ZEN Blue Lite 2.3 and ImageJ_V1.8 software.

### 2.5. Quantitative Real-Time PCR (qRT-PCR) Assays

After sacrifice, zebrafish livers were collected for mRNA extracting using TRIzol reagent (Thermo Fisher Scientific, Waltham, MA, USA). Each qRT-PCR reaction (20 µL) contained 10 µL of 2× SYBR Green Master Mix (Yeasen Biotechnology, Shanghai, China), 0.5 µL of forward and reverse primers, 1 µL of cDNA, and 8 µL of RNase-free water. Three livers were pooled as a replicate (*n* = 3). The specific primer sequences for qRT-PCR are listed in Table S1 of Supplementary Materials. Relative gene expression levels were calculated using the 2^−ΔΔCt^ method, with β-actin as the reference gene. The primer pairs were listed in Tables S1 and S2 in Supplementary Materials.

### 2.6. Liver Proteome Analysis

Liver tissues from three individuals were pooled to create each biological replicate (*n* = 3). Total protein was extracted according to the previous protocol with minor modification [26]. Samples were lysed with SDT buffer containing 4% SDS, 100 mM Tris-HCl, 100 mM NaCl, 1% DTT (*v*/*v*), and pH7.6. The lysate was vortexed, sonicated for 5 min on ice, heated at 95 °C for 10 min, and then cooled on ice for 2 min. Following centrifugation at 12,000 g for 15 min at 4 °C, the supernatant was then collected for alkylation using sufficient iodoacetamide (IAM) (Merck, Darmstadt, Germany). After quality examination, protein was digested overnight using trypsin (Promega, Madison, WI, USA) at 37 °C and desalted using a C18 cartridge. The lyophilized peptides were reconstituted in 100 µL of 0.1 M TEAB buffer and labeled using TMT^®^ Mass Tagging Kits and Reagents (Thermo Fisher Scientific Inc., Waltham, MA, USA) according to the manufacturer’s instructions. Peptide separation was performed using an EASY-nLC™ 1200 UHPLC system (Thermo Fisher Scientific Inc., Waltham, MA, USA) and mass spectrometric analysis was conducted on a Q Exactive™ HF-X mass spectrometer (Thermo Fisher Scientific Inc., Waltham, MA, USA) equipped with a Nanospray Flex™ (ESI) (Thermo Fisher Scientific Inc., Waltham, MA, USA) source. The instrument was operated in data-dependent acquisition (DDA) mode with the full MS scan range of *m*/*z* 407–1500 and a resolution of 60,000 (at *m*/*z* 200). Raw data files were processed using Proteome Discoverer (PD) software V3.3. Principal Component Analysis (PCA) was performed to visualize global protein expression patterns and sample clustering. Differentially expressed proteins were identified based on a Student’s *t*-test (*p* < 0.05) and a fold change (FC) threshold of |log2FC| > 1. Differentially expressed proteins were further analyzed using volcano plots, hierarchical clustering heatmaps, and enrichment analyses.

### 2.7. Statistical Analyses

Data are presented as mean ± standard error (SEM). The homogeneity of variance was assessed using Levene’s test. One-way analysis of variance (ANOVA) was performed for group comparisons, followed by Tukey’s post hoc test for multiple comparisons. Statistical analyses were conducted using SPSS 26 (IBM, Armonk, NY, USA), and a *p*-value of <0.05 was considered statistically significant.

## 3. Results

### 3.1. TBPH Exposure Unbalances the Homeostasis in Zebrafish Liver

**Figure 1:**
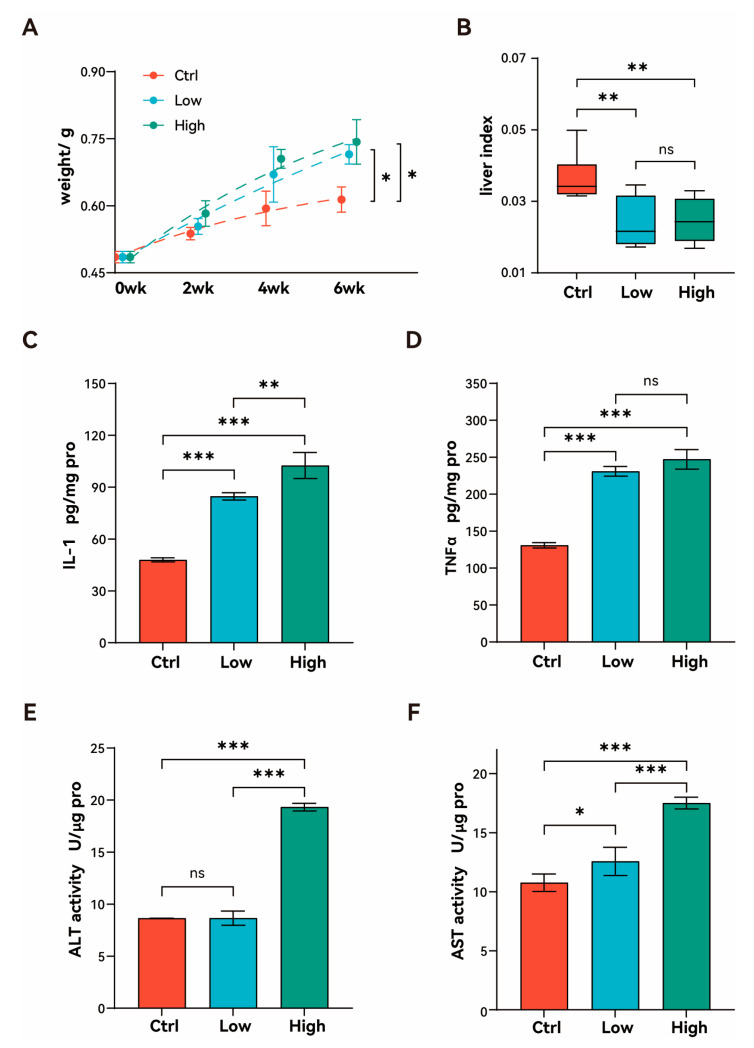
Effects of TBPH exposure on growth performance and liver health. (**A**) Body weight changes in zebrafish exposed to different TBPH concentrations. (**B**) Liver index of zebrafish after six weeks of TBPH exposure. (**C**,**D**) Hepatic IL-1 (**C**) and TNF-α (**D**) concentrations in zebrafish following six weeks of TBPH exposure. (**E**,**F**) Hepatic ALT (**E**) and AST (**F**) activities in zebrafish after six weeks of TBPH exposure. Ctrl: control group (0 μM TBPH); Low: low-dose group (0.02 μM TBPH); High: high-dose group (2 μM TBPH). *, **, and *** indicate *p* < 0.05, *p* < 0.01, and *p* < 0.001, respectively; ns indicates no significant difference (*p* > 0.05).

After 6 weeks of exposure to TBPH, the body weight of zebrafish in both the low- and high-concentration exposure groups increased significantly compared to the control group (*p* < 0.05; Figure 1A). However, there was no statistical difference between these two exposure groups. Furthermore, the liver index was also markedly influenced by TBPH exposure. Both the low- and high-concentration groups showed a significant decrease in liver index relative to the control group (*p* < 0.01; Figure 1B). In the aspects of hepatic proinflammatory cytokines, TBPH exposure induced a significant elevation in IL-1 levels (*p* < 0.001; Figure 1C). Moreover, IL-1 concentration exhibited a progressive increase from the low- to the high-concentration group, demonstrating a clear dose-dependent relationship (*p* < 0.01; Figure 1C). Similarly, TNFα levels were significantly elevated in both exposure groups compared to the control group (*p* < 0.001); no significant difference was observed between these two exposure groups (Figure 1D). Assessment of hepatic enzyme activities revealed that ALT activity remained at baseline levels in the low-concentration group, but significantly increased in the high-concentration group compared to both the control and low-concentration group (*p* < 0.001; Figure 1E). Meanwhile AST showed a significant increase in the low-concentration group (*p* < 0.05), with a further significant elevation in the high-concentration group compared to both the control and low-concentration group (*p* < 0.0001; Figure 1F).

### 3.2. TBPH Exposure Leads to Steatosis and Fibrosis in Zebrafish Liver

**Figure 2:**
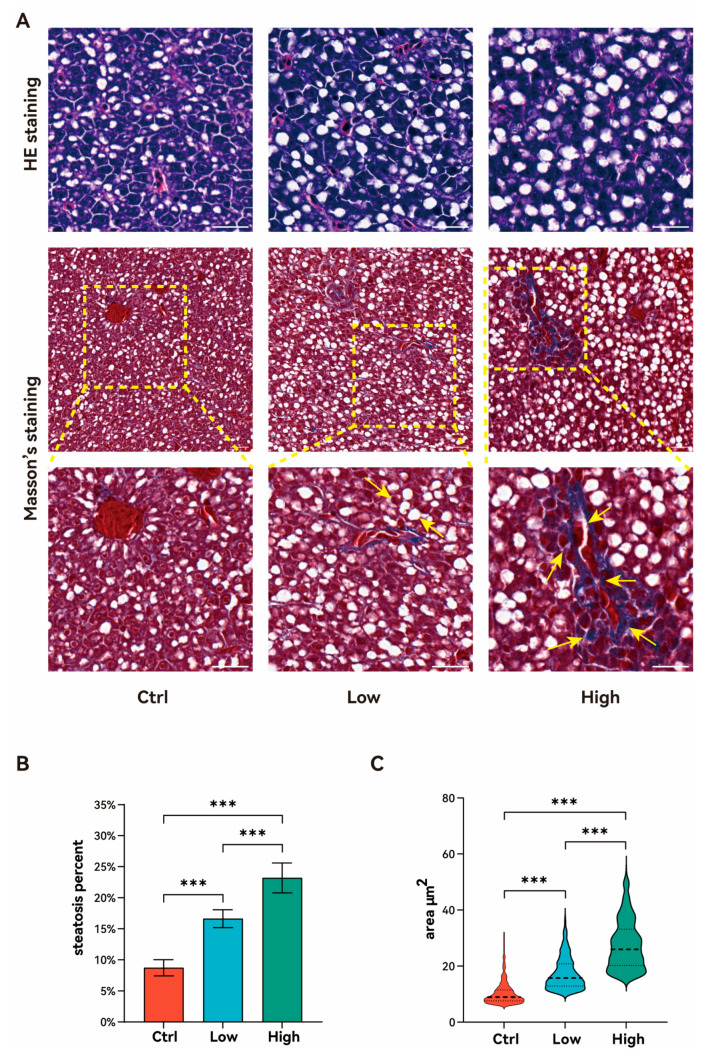
Effects of TBPH exposure on hepatic histopathological alterations. (**A**) Representative images of liver sections subjected to hematoxylin–eosin (HE) staining (steatosis) and Masson’s trichrome staining (fibrosis) after six weeks of TBPH exposure. Scale bar = 20 μm. The yellow dashed box indicates the magnified region, and yellow arrows denote areas of collagen deposition. (**B**) Hepatic steatosis percentage in zebrafish after six weeks of TBPH exposure. (**C**) Average lipid vacuole area in zebrafish hepatocytes following six weeks of TBPH exposure. Black dashed line means the median. Ctrl: control group (0 μM TBPH); Low: low-dose group (0.02 μM TBPH); High: high-dose group (2 μM TBPH). *** indicates *p* < 0.001.

HE and Masson’s trichrome staining were used to detect liver morphological alternations following TBPH exposure. HE staining revealed marked lipid accumulation in the hepatocytes in both exposure groups (Figure 2A), with a notable increase in the density and diameter of the lipid droplets (LDs). The hepatic steatosis percentage of the liver was significantly elevated after six weeks of low-concentration TBPH exposure compared to the control group (*p* < 0.001; Figure 2B). Moreover, the degree of steatosis percent exhibited a clear dose-dependent increase with the high-concentration group showing significantly higher increases than the low-concentration group (Figure 2B). Consistently, the mean diameter of the hepatocellular LDs also increased in a dose-dependent manner following a six-week TBPH exposure (Figure 2C). Masson’s trichrome staining further demonstrated that collagen deposition (blue staining) remained localized within the portal tract in the low-concentration group. But the collagen-positive area extended beyond the portal tract in the high-concentration group (Figure 2A), indicating the early onset of hepatic fibrosis.

### 3.3. TBPH Affects the Lipid Metabolism in Zebrafish Liver

**Figure 3:**
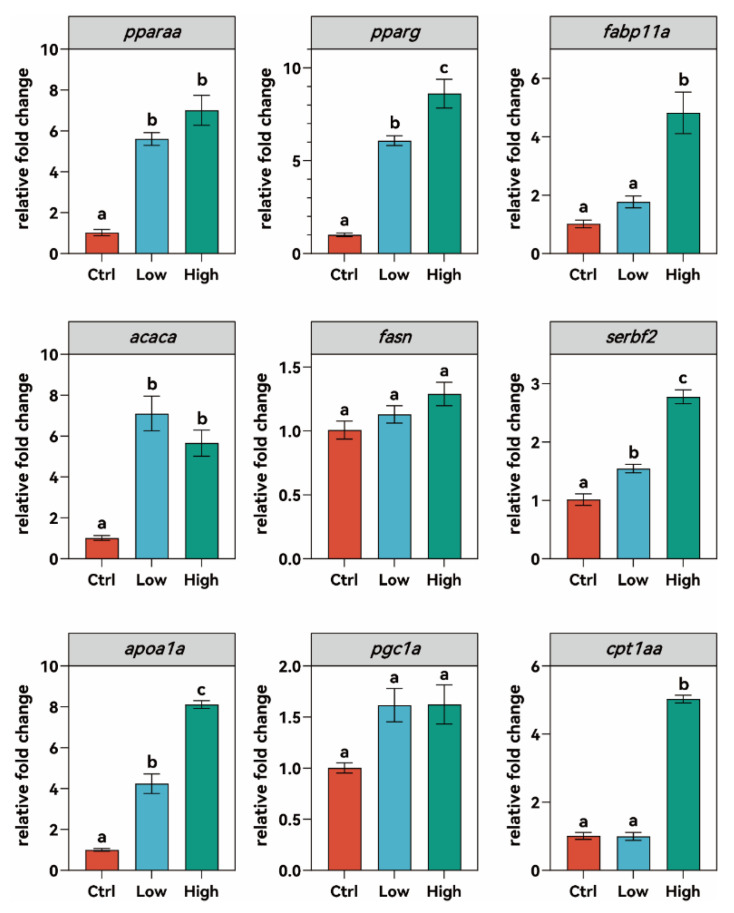
Hepatic expression of lipid metabolism–related genes in zebrafish after six weeks of TBPH exposure. Ctrl: control group (0 μM TBPH); Low: low-dose group (0.02 μM TBPH); High: high-dose group (2 μM TBPH). Bars marked with different letters represent statistically significant differences (*p* < 0.05).

After six weeks of TBPH exposure, zebrafish exhibited significant disruptions in hepatic lipid metabolism (Figure 3). Gene expression analysis showed that TBPH exposure markedly upregulated the expression of hepatic *pparaa* and *pparg*, two key members of the peroxisome proliferator-activated receptor (PPAR) family, compared to the control group (*p* < 0.05). Hepatic *pparaa* expression did not differ significantly between the low- and high-concentration groups, while *pparg* expression increased a clear dose-dependent manner. The expression of *fabp11a* (fatty acid-binding protein 11a) remained at baseline levels in the low-concentration group but was significantly upregulated in the high-concentration group (*p* < 0.01). Similarly to *pparg*, *acaca* (acetyl-CoA carboxylase alpha) expression was significantly upregulated in both exposure groups (*p* < 0.05), while there was no significantly difference between these two exposure groups. In contrast, *fasn* (fatty acid synthase) and *pgc1a* (peroxisome proliferator-activated receptor gamma coactivator 1 alpha) levels remained unaltered following TBPH exposure. Furthermore, *srebf2* (sterol regulatory element-binding transcription factor 2) and *apoa1a* (apolipoprotein A-1a) transcripts were both significantly upregulated in a dose-dependent manner, with expression levels in the high-concentration group significantly higher than those in the control and low-concentration groups (*p* < 0.05). The expression of *cpt1aa* (carnitine palmitoyltransferase 1Aa) was unaffected in the low-concentration group but was significantly increased in the high-concentration group compared to both the control and low-concentration groups (*p* < 0.05).

### 3.4. TBPH Stimulate the LDs Biogenesis In Vitro

**Figure 4:**
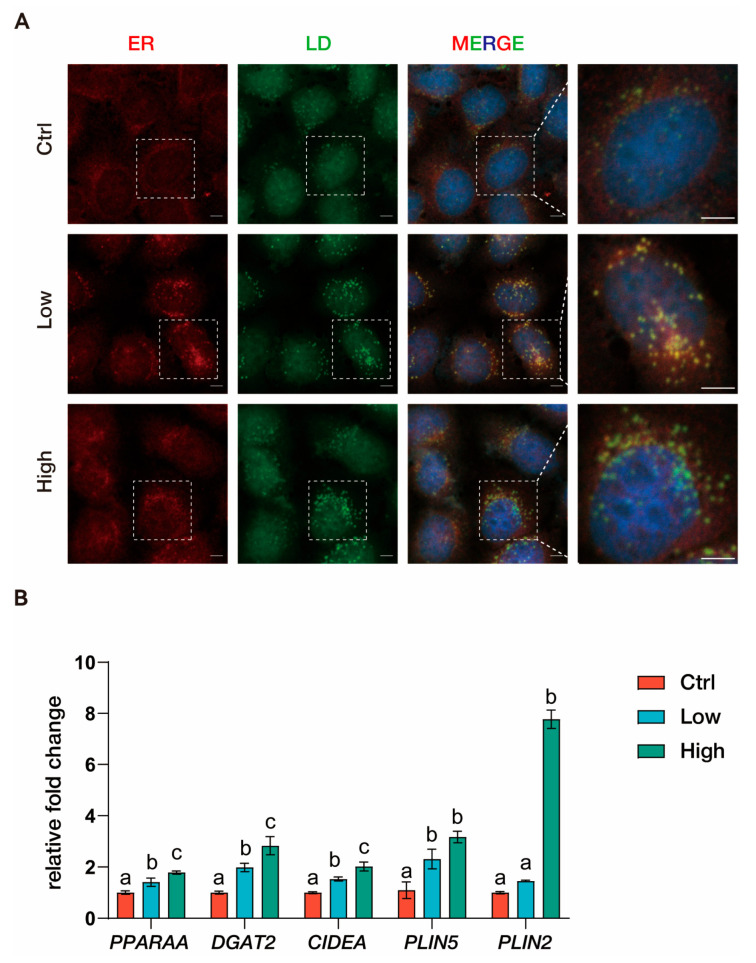
Lipid droplet biogenesis and gene expression in HepG2 cells exposed to TBPH. (**A**) Representative immunofluorescence images of HepG2 cells following TBPH exposure. Nuclei were stained with DAPI (blue), lipid droplets (LD) with LipidSpot 488 (green), and the endoplasmic reticulum (ER) was visualized using ERp57 immunofluorescence (red). The white dashed box indicates the magnified region. Scale bar = 5 μm. (**B**) Relative expression levels of lipid droplets related genes in HepG2 cells after TBPH exposure. Ctrl: control group (0 nM TBPH); Low: low-dose group (0.2 nM TBPH); High: high-dose group (2 nM TBPH). Bars marked with different letters represent denote statistically significant differences (*p* < 0.05).

HepG2 cells were employed to investigate TBPH-induced lipid droplet accumulation. Immunofluorescence imaging revealed a dose-dependent increase in lipid droplet accumulation in TBPH-exposed HepG2 cells. In untreated controls, the ER displayed a well-defined morphology with basal lipid droplet formation. The low-concentration group induced the initial accumulation of small LDs adjacent to the ER. At high TBPH concentrations, LDs exhibited a pronounced increase in both number and size, with evident co-localization to the ER. Real-time qPCR analysis revealed alterations in transcript levels in HepG2 cells following exposure to TBPH (Figure 4). The expression of *PPARA* (Peroxisome Proliferator-Activated Receptor Alpha), *DGAT2* (Diacylglycerol O-Acyltransferase 2), and *CIDEA* (Cell Death-Inducing DFFA-Like Effector A) exhibited significant dose-dependent increases upon TBPH exposure, with markedly higher expression levels in the high-concentration group compared with both the low-concentration and control groups. The expression of *PLIN5* (Perilipin 5) was upregulated in both the low- and high-concentration groups, with no significant difference between them. In contrast, the transcription level of *PLIN2* (Perilipin 2) remained unchanged in the low-concentration group relative to the control but showed a significant increase in the high-concentration group compared with both the low and control groups (*p* < 0.001).

In the control group, minimal lipid droplet formation was observed, and the ER remains well-organized without significant lipid accumulation. In the low-dose TBPH group, small LDs began to form, suggesting an early response to TBPH exposure. In the high-dose TBPH group, there was a dramatic increase in the number and size of LDs, with a notable co-localization of LDs with the ER, indicating enhanced lipid droplet biogenesis. This result revealed that TBPH exposure triggers the accumulation of LDs, which may be linked to dysregulated lipid metabolism or stress responses, particularly at higher concentrations. The close association of LDs with the ER at high TBPH levels further suggested that the ER plays a key role in lipid droplet formation under these conditions.

### 3.5. TBPH Stimulate the Fibrosis Progress Through Mfap4 Signal Pathway

**Figure 5:**
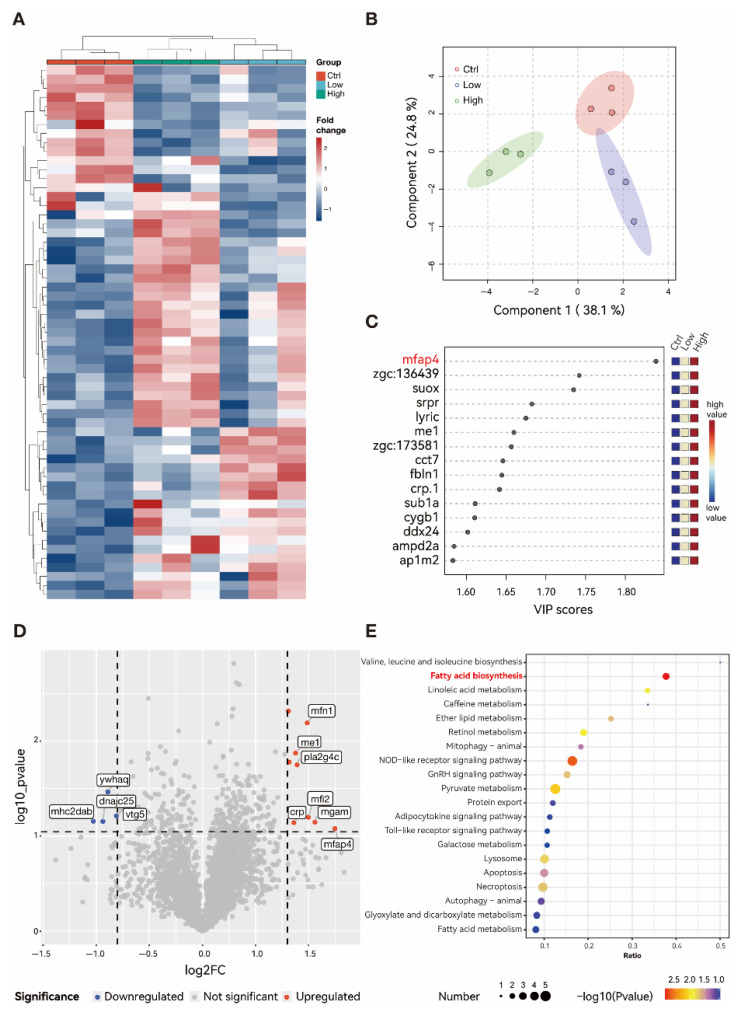
Hepatic proteomic profiling of adult zebrafish following chronic exposure to TBPH (6 weeks). (**A**) Hierarchical clustering heatmap of differentially expressed proteins across all experimental groups. (**B**) Principal Component Analysis (PCA) score plot based on global protein expression profiles. Different color areas mean PCA confidence intervals of each group. (**C**) Variable Importance in the Projection (VIP) scores derived from PLS-DA, identifying the top contributing proteins in the high-concentration group versus the control group. Red marker indicates proteins with the highest VIP scores. (**D**) Volcano plot illustrating significantly upregulated (red) and downregulated (blue) proteins in the high-concentration group compared to the control. Black dashed lines indicate the thresholds for fold change and *p*-value. (**E**) KEGG pathway enrichment analysis of differentially expressed proteins in the high-concentration group compared to the control. Red marker indicates critical signaling pathway involved. Ctrl: control group (0 μM TBPH); Low: low-dose group (0.02 μM TBPH); High: high-dose group (2 μM TBPH).

The hepatic proteome characterized key drivers of fibrosis and lipid metabolic disorder. Unsupervised hierarchical clustering analysis revealed that the differentially expressed proteins in both high- and low-concentration groups exhibited consistent expression patterns and were clearly distinct from those in the control group (Figure 5A). This clustering pattern was further corroborated by principal component analysis (PCA), where the low-concentration group clustered in close proximity to the control group, whereas the high-concentration group showed the greatest separation from the other two groups (Figure 5B). These findings suggest a dose-dependent relationship between exposure concentration and alterations in protein expression. Multivariate statistical analysis using Partial Least Squares-Discriminant Analysis (PLS-DA) identified Mfap4 (microfibril-associated protein 4) as the protein with the highest Variable Importance in the Projection (VIP) score (VIP > 1.8) in the high-concentration group compared to controls (Figure 5C). MFAP4 is a key driver of hepatic fibrosis, and its significant upregulation is highly consistent with the hepatocellular fibrotic degeneration observed in the histological examination of the high-concentration group. Volcano plot analysis further demonstrated that Mfap4, Mfn1 (Mitofusin 1), and Mfi2 (melanotransferrin) were the most significantly upregulated proteins in the high-concentration group (Figure 5D). Mfn1 is a GTPase located on the outer membrane of mitochondria involving the processes of fusion and maintaining the potential of mitochondrial members. Meanwhile the elevated expression of Mfn1 implies mitochondrial dysfunction, which may disrupt energy metabolism and induce oxidative stress, subsequently leading to aberrant lipid metabolism and inflammatory responses. Furthermore, KEGG pathway enrichment analysis of the differentially expressed proteins between the high-concentration and control groups highlighted a significant enrichment of lipid metabolism-related pathways, including fatty acid biosynthesis and linoleic acid metabolism (Figure 5E). These findings align with the observed pathological phenotypes of hepatic lipid accumulation, steatosis, and fibrosis, suggesting that the exposure may accelerate liver injury progression by disrupting lipid metabolic homeostasis.

## 4. Discussion

The global prevalence of MASLD has risen alarmingly over the past three decades, driven primarily by the pandemics of obesity and type 2 diabetes mellitus (T2DM) [27,28]. Aside from obesity and T2DM, environmental factors may predispose to progressive liver disease [29,30]. Among them, the widespread TBPH detected in biotic and abiotic mediums has raised concerns on a potential health risk in humans [31,32,33]. In our previous studies, we found that TBPH exposure can disturb the lipid metabolism in the early development stage [7,34] and also the adult stage [8,35] in zebrafish through the *pparg* signaling pathway and also foxo1-mediated lipophagy. But the long-term consequences remained under-explored. The present study bridges this gap, revealing that chronic TBPH exposure does not merely induce steatosis but exacerbates the transition to metabolic dysfunction-associated steatohepatitis (MASH) and fibrosis, disrupting physiological homeostasis far beyond the effects of acute exposure.

The transition from simple steatosis to MASH is often precipitated by lipotoxicity and chronic inflammation [36]. In this study, the significant elevation of ALT and AST activity and proinflammatory cytokines (TNFα, IL-1) confirms that TBPH exposure induces hepatocellular injury [37]. Exposure to various chemical pollutants can activate the hepatic PPAR gamma signal pathway [38]. This activation may promote excessive lipid accumulation, ultimately leading to lipid toxicity and resulted in chronic liver inflammation [39]. We observed that TBPH exposure simultaneously activated both hepatic lipid synthesis and oxidation signaling pathways. The upregulation of the *pparg* axis and its downstream target *acaca* suggests that de novo lipogenesis was the dominant driver [40]. Acaca catalyzes the critical conversion of acetyl-CoA to malonyl-CoA, providing the substrate for fatty acid biosynthesis. The significant upregulated expression of *acaca* in the exposure groups in this study confirmed the stimulation of hepatic de novo fatty acid biogenesis in zebrafish upon TBPH exposure, which is similar to that in the experiment of the palmitic acid-induced MASLD in zebrafish [41]. Furthermore, TBPH exposure induced robust hepatic lipid accumulation, resulting in severe steatosis that progressed in a strictly dose-dependent manner. Although genes associated with both fatty acid oxidation (*cpt1aa*) and transport (*fabp11a*, *apoa1a*) were upregulated, the persistence of severe hepatic steatosis and lipid droplet expansion suggests that TBPH-induced lipogenesis likely exceeds the hepatocellular capacity for lipid oxidation and secretion [42,43]. Future studies incorporating direct metabolic flux and mitochondrial respiration assays are warranted to functionally confirm this metabolic imbalance.

In vitro experiments in HepG2 cells provide high-resolution insight into the cellular dynamics of this accumulation. TBPH exposure stimulated LD biogenesis in a concentration-dependent manner, characterized by the upregulation of DGAT2, PLIN2, and PLIN5. DGAT2 is essential for triglyceride synthesis at the ER membrane. LDs are highly dynamic organelles critical for the orchestration of lipid metabolism and the maintenance of cellular homeostasis across physiological and pathological states [44,45]. Immunofluorescence imaging revealed that high-concentration TBPH exposure induced the formation of numerous enlarged LDs and co-localization with the ER. This ER-LD co-localization and the upregulation of CIDEA facilitated lipid transfer and droplet fusion, promoting the formation of macrovesicular steatosis [46,47]. This in vitro experiment provided direct evidence that TBPH stimulate the LD biogenesis and development, thereby driving the excessive lipid storage and steatosis observed in the zebrafish model.

In addition, another pivotal finding is the hepatic fibrosis in the high-concentration exposure group. Proteomic analysis identified Mfap4 as the top-ranking differentially expressed protein among the high-concentration group and the control. Mfap4 is an extracellular matrix protein located in elastic fibers which has been proven as a key trigger of liver fibrosis [48,49]. Under pathological conditions, Mfap4 is secreted by activated hepatic stellate cells, promotes their motility and enhances the apoptosis resistance through a self-sustaining feedback loop, thus maintaining the hepatic stellate cell activation [50]. This sustained activation functionally maps onto classical fibrotic cascades, driving the transdifferentiation of HSCs into α-SMA-expressing myofibroblasts, which are ultimately responsible for the massive deposition of Collagen I and other structural extracellular matrix proteins [51]. The activation of downstream NF-κB signaling pathways not only perpetuates the survival of fibrogenic cells by apoptosis resistance but also amplifies the proinflammatory signal (IL-1/TNFα), resulting in collagen deposition and fibrosis progress [52]. This signaling cascade amplifies proinflammatory signals, perpetuating collagen deposition and fibrogenesis. Furthermore, our proteomic data illuminates the critical role of mitochondrial remodeling. The upregulation of Mfn1 suggested a pathological alteration in mitochondrial dynamics [53]. While mitochondrial fusion is typically a response to metabolic stress to maximize oxidative capacity, in the context of lipid overload, upregulated Mfn1 may facilitate the tethering of mitochondria to LDs [54,55]. This contact provided ATP to support the enzymes involved in triglyceride synthesis and LD expansion [56]. Meanwhile, oxidative stress induced by mitochondrial dysfunction can activate latent TGF-β, further stimulating HSCs and driving fibrotic outcomes [57,58].

Collectively, these results define a comprehensive adverse outcome pathway for TBPH-induced hepatotoxicity. TBPH exposure activated the *pparg* signal pathway, driving extensive de novo lipogenesis that exceeds the hepatocyte’s adaptive threshold. This unresolved lipid burden triggers chronic inflammation and activates the Mfap4 signaling pathway, which promoted hepatic stellate cell activation and collagen deposition. Ultimately, this cascade culminates in the hepatic fibrosis process. These findings underscore the potential of TBPH to act as a fibrosis stimulator, posing significant risks for the progression of MASLD in aquatic organisms and potentially humans. Ultimately, our comprehensive proteomic profiling provides robust, functional-level evidence of this Mfap4-driven fibrotic cascade. Therefore, we propose that Mfap4 serves as a promising exploratory candidate linking initial TBPH-induced lipid dysregulation to subsequent fibrotic remodeling. The precise molecular mechanisms by which Mfap4-related pathways mediate this TBPH-induced fibrotic response warrant dedicated functional investigation and will be the primary focus of our future studies.

## 5. Conclusions

This study provides compelling evidence that chronic exposure to TBPH acts as a potent environmental disruptor of hepatic homeostasis, driving the progression from simple steatosis to fibrotic steatohepatitis (MASH) in zebrafish. While previous reports have established the link between TBPH and lipid dysregulation, our findings elucidate a distinct mechanism where long-term exposure significantly exacerbates liver injury beyond metabolic adaptation.

## Data Availability

The data presented in this study are available upon request from the corresponding author.

## Supplementary Materials

The following supporting information can be downloaded at: https://www.mdpi.com/article/10.3390/biology15060463/s1, Table S1: qRT-PCR primer pairs used in zebrafish; Table S2: qRT-PCR primer pairs used in HepG2.
