# Supplementary material for: Chronic TBPH Exposure Drives the Transition from Steatosis to Hepatic Fibrosis via Lipid Droplet Dysregulation in Zebrafish
Source: Biology (Basel). 2026 Mar 13;15(6):463. doi: 10.3390/biology15060463 (PMC13023650; doi:10.3390/biology15060463)
Supplement: Supplementary file 1 [file biology-15-00463-s001.zip › biology-4170750-supplementary.pdf]

Supplementary Tables

Table S1. qRT-PCR primer pairs used in zebrafish.

Gene Name	Accession No.	Forward Primer (5'-3')	Reverse Primer (5'-3')
acaca	XM_017356109.2	GGGCACAAAGACCGACAGAT	GCCTGGCGAAACATTTCTGG
actb	AF025305.1	ACAGGGAAAAGATGACACAGATCA	CAGCCTGGATGGCAACGTA
apoa1a	NM_131128.1	TAAGCTGACCGAGCGTCTTG	TCTGTGCGAATGTGGTCCTC
cpt1aa	XM_005166474.4	GCGGTCTTGCACTACAGAGT	CCCCTGCTGTAGAGGAGTCT
fabp11a	NM_001004682.1	AGCACCTTCAAAACCACCGA	TCCCAGGTCTGTTTCTGCAC
fasn	XM_682295.5	ATGGAGTTTTCAGGGCGAG	GGGAATAATATGCGGTGGC
pgc1a	XM_017357140.2	AGATGGGGACGTGACCAATG	GGGGTTTTCTGTCTTGGCAAC
pparaa	NM_001161333.1	CTGGGAGACCCGATTA	TGCTGGCTGAGAACAC
pparg	NM_131467.1	CTGCCGCATACACAAGAAGA	TCACGTCACTGGAGAACTCG
srebf2	NM_001089466	CACTCACACAAGCACACACG	ACCTGGTTCTGGATGAATCG

Table S2. qRT-PCR primer pairs used in HepG2

Gene Name	Accession No.	Forward Primer (5'-3')	Reverse Primer (5'-3')
ACTB	NM_001101.5	GTCCACCGCAAATGCTTCTA	TGCTGTCACCTTCACCGTTC
CIDEA	NM_001279.4	CTCATCAGGCCCCTGACATT	CGGCATCCACTTCTGTCCTT
DGAT2	NM_001253891.2	TCGAGACTACTTTCCCATCCA	GGTGGTATCCAAAGATATAGTTCCTG
PLIN2	NM_001122.4	TTGCAGTTGCCAATACCTATGC	CCAGTCACAGTAGTCGTCACA
PLIN5	NM_001013706.3	AAGGCCCTGAAGTGGGTTC	GCATGTGGTCTATCAGCTCCA
PPARA	NM_001393946.1	TTCGCAATCCATCGGCGAG	CCACAGGATAAGTCACCGAGG
